# Decrease of Perivascular Adipose Tissue Browning Is Associated With Vascular Dysfunction in Spontaneous Hypertensive Rats During Aging

**DOI:** 10.3389/fphys.2018.00400

**Published:** 2018-04-18

**Authors:** Ling-Ran Kong, Yan-Ping Zhou, Dong-Rui Chen, Cheng-Chao Ruan, Ping-Jin Gao

**Affiliations:** ^1^State Key Laboratory of Medical Genomics, Shanghai Key Laboratory of Hypertension, Department of Hypertension at Ruijin Hospital and Shanghai Institute of Hypertension, Shanghai Jiao Tong University School of Medicine, Shanghai, China; ^2^Key Laboratory of Stem Cell Biology, Institute of Health Sciences, Shanghai Jiao Tong University School of Medicine and Shanghai Institutes for Biological Sciences, Chinese Academy of Sciences, Shanghai, China

**Keywords:** PVAT, browning, adenosine, vasodilation, SHR

## Abstract

Functional perivascular adipose tissue (PVAT) is necessary to maintain vascular physiology through both mechanical support and endocrine or paracrine ways. PVAT shows a brown adipose tissue (BAT)-like feature and the browning level of PVAT is dependent on the anatomic location and species. However, it is not clear whether PVAT browning is involved in the vascular tone regulation in spontaneously hypertensive rats (SHRs). In the present study, we aimed to illustrate the effect of aging on PVAT browning and subsequent vasomotor reaction in SHRs. Herein we utilized histological staining and western blot to detect the characteristics of thoracic PVAT (tPVAT) in 8-week-old and 16-week-old SHR and Wistar-Kyoto (WKY) rats. We also detected vascular reactivity analysis to determine the effect of tPVAT on vasomotor reaction during aging. The results showed that tPVAT had a similar phenotype to BAT, including smaller adipocyte size and positive uncoupling protein-1 (UCP1) staining. Interestingly, the tPVAT of 8-week-old SHR showed increased BAT phenotypic marker expression compared to WKY, whereas the browning level of tPVAT had a more dramatic decrease from 8 to 16 weeks of age in SHR than age-matched WKY rats. The vasodilation effect of tPVAT on aortas had no significant difference in 8-week-old WKY and SHR, whereas this effect is obviously decreased in 16-week-old SHR compared to WKY. In contrast, tPVAT showed a similar vasoconstriction effect in 8- or 16-week-old WKY and SHR rats. Moreover, we identified an important vasodilator adenosine, which regulates adipocyte browning and may be a potential PVAT-derived relaxing factor. Adenosine is dramatically decreased from 8 to 16 weeks of age in the tPVAT of SHR. In summary, aging is associated with a decrease of tPVAT browning and adenosine production in SHR rats. These may result in attenuated vasodilation effect of the tPVAT in SHR during aging.

## Introduction

Perivascular adipose tissue (PVAT) is a structure surrounding most of the blood vessels, which plays vital roles in vascular physiopathology (Brown et al., 2014). As an essential part of the vasculature, PVAT is involved in various vascular diseases, including atherosclerosis, aneurysm, and hypertension (Takemori et al., 2007; Verhagen and Visseren, 2011; Sakaue et al., 2017). It is well established that adipose tissue includes brown adipose tissue (BAT) and white adipose tissue (WAT). WAT predominately serves to store energy. In contrast, BAT dissipates energy through uncoupled respiration and thermogenesis, and protects against metabolic disorders (Kozak, 2010). PVAT shows a BAT-like feature and the browning level of PVAT is dependent on the anatomic location and species (Britton and Fox, 2011). Thoracic PVAT (tPVAT) shares structural, genetic, and proteomics features with BAT (Chang et al., 2012), including several small multilocular lipid droplets and abundant mitochondria. Previous studies showed that the tPVAT of spontaneously hypertensive rats (SHRs) has structural and functional changes compared with Wistar-Kyoto (WKY) rats (Galvez et al., 2006). Hypertension is a highly prevalent disease with advancing age. Aging is also associated with whole-body adipose tissue redistribution, with a relative loss of BAT in interscapular area and an accumulation of WAT in the trunk and visceral areas (Schosserer et al., 2017). Several reports suggest that aging induces superoxide production and inhibits adiponectin expression in PVAT, leading to arterial stiffening (Fleenor et al., 2014; Agabiti-Rosei et al., 2017). However, little is known about the changes in browning level in the tPVAT of SHR rats during aging.

As an active endocrine organ, PVAT not only releases abundant adipokines to regulate vascular functions, it can also produce small molecular active substances such as nitric oxide and reactive oxygen species, which could directly regulate vascular contraction and relaxation (Victorio et al., 2016). For example, compared to WKY, the PVAT of SHR releases significantly less methyl palmitate, these contribute to vascular tone regulation and pathogenesis of hypertension (Lee et al., 2011). However, there are many other PVAT derivatives which may play important roles under both physiological and pathological conditions. Adenosine, a vasorelaxant substance, is an endogenous purine nucleoside comprised of adenine and ribose joined by a glycosidic bond (Borea et al., 2016). The terminal synthesis of extracellular adenosine is mediated by CD73 (ecto-5-nucleotidase), following activity of CD39 (nucleoside triphosphate dephosphorylase). It is well known that adenosine formed by CD73 plays a key role in the regulation of cardiac inflammation and fibrosis (Quast et al., 2017). Adenosine could be produced by adipocytes through activating CD73 (Burghoff et al., 2013). It is a novel activator of brown and beige fat, and abundantly exists in BAT (Gnad et al., 2014).Therefore, we speculated that adenosine might be a PVAT-derived relaxing factor (PVRF) and we hypothesized that the aging-reduced browning level of PVAT is associated with decrease of adenosine production, which is associated with the regulation of vasomotor reaction in SHR rats.

## Materials and Methods

### Animals

Experiments were conducted in 8-week-old and 16-week-old male WKY and SHR. The rats were provided by Shanghai SLAC Laboratory Animal Company. Body weight was measured in conscious animals, then the rats were anesthetized with intraperitoneal injection of pentobarbital (5 mg/100 g) and sacrificed. The tPVAT, subcutaneous WAT (sWAT), and interscapular BAT (iBAT) were dissected out and immersed in liquid nitrogen immediately for subsequent analysis. Animals were housed under standard laboratory conditions and had a free access to drinking water and food. All animal procedures were approved in accordance with institutional guidelines established by the Committee of Ethics on Animal Experiments at the Shanghai Jiao Tong University School of Medicine.

### Blood Pressure and Body Weight Measurement

Blood pressures were measured as described previously (Jin et al., 2017). Briefly, systolic and diastolic blood pressure (SBP and DBP) was measured in conscious rats by the tail-cuff method (BP-2000, Visitech Systems, United States) and the average of at least 20 readings was calculated. Blood pressure and body weight were measured with 10 rats in each group.

### Histological Analysis

For hematoxylin/eosin (HE) staining, thoracic aortas with intact surrounding tPVAT were fixed in 10% formaldehyde at 4°C for 48 h, washed in water, and embedded in paraffin. Cross-sections (5 μm) were deparaffinized in xylene, rehydrated, and washed in physiological basic salt solution (PBS). PVAT cross-sectional area was measured by HE staining as previously described (Ma et al., 2010). Images were captured using a Carl Zeiss Axio Imager M2 microscope (Carl Zeiss Corporation, Germany). Morphometric analysis was performed with software Image-Pro Plus (Media Cybernetics, United States) by counting the number of adipocytes in the same area (×200 magnification image) of four animals in each group as previously described (Sheng et al., 2016).

To identify brown adipocytes from white adipocytes in the tPVAT, we performed UCP1 immunohistochemical staining in PVAT of SHR and WKY rats. Endogenous peroxidase activity was quenched in hydrogen peroxide for 10–15 min and then the sections were washed three times in PBS buffer. Sections were then incubated with the primary antibody, anti-rabbit UCP1 antibody (1:100 dilution) (Abcam, ab23841) at 4°C overnight, and then washed in PBS and incubated with the secondary antibody (1:200 dilution) (Proteintech, SA00001-2). Tissue sections were counterstained with Mayer’s hematoxylin solution, rinsed in running tap water, dehydrated, and mounted with permanent mounting medium. Control sections were incubated with PBS in place of the primary antibody. Sections with primary antibody omitted in staining were used as controls. UCP1-positive adipocyte area was counted under microscope with the software Image-Pro Plus (Media Cybernetics, United States).

### Western Blot Analysis

The frozen tPVAT, sWAT, and iBAT tissues were lysed in RIPA buffer (Merck Millipore, 20-188) containing 1% protease inhibitor cocktail (Biotool, B14002). The protein extractions were collected for western blot as previously described (Harms et al., 2014). The primary antibodies were as follows: anti-UCP1 (1:1000 dilution) (Abcam, ab23841), anti-peroxisome proliferator-activated receptor-γ (PPARγ, 1:1000 dilution) (Santa Cruz, sc-7273), anti-PPARγ-coactivator 1-α (PGC1α, 1:1000 dilution) (Santa Cruz, sc-13067), anti-CD73 (1:1000 dilution) (Abcam, ab108248), and GAPDH monoclonal antibody (1:4000 dilution) (Proteintech, HRP-6004). Immunoreactive bands were highlighted by electrochemiluminescence (ECL) technology and quantified using imaging software Quantity One (Bio-Rad Laboratory, Spain).

### Vascular Reactivity in Thoracic Arteries

Thoracic aorta from 8-week-old WKY was isolated and the PVAT was carefully removed. The aorta was cut into 2–3 mm rings and put in cell culture plate. tPVAT (0.3 g) was obtained from 8-week-old or 16-week-old donor WKY and SHR under anesthesia and snipped into small pieces. The rings were incubated with tPVAT in Dulbecco’s Modified Eagle’s Medium (DMEM) for 12 h in 37°C. Then each aorta was fixed on the isometric force transducer (Danish Myo Technology Model 610 M, Denmark) in a 5 ml organ bath, aerating with 95% O_2_ and 5% CO_2_ under an initial resting tension of 2.5 mN (Zhou et al., 2014). Lumen diameter was recorded in a Power Lab/8sp data acquisition system (A.D. Instruments, Castle Hill, Australia). Aorta was incubated in oxygenated Krebs’ medium (containing: KCl 4.7 mM, NaCl 118 mM, CaCl_2_ 2.5 mM, KH_2_PO_4_ 1.2 mM, MgSO_4_ 1.2 mM, glucose 11 mM, and NaHCO_3_, 25 mM) for 60 min at pH 7.4 and 37°C. The contractility was tested three times by high K^+^ mediums (60 mM KCl) to stabilize the contraction. A dose–response curve of phenylephrine (PHE, 0.01–100 μM) was performed to assess the vasoconstriction response and cumulative concentration–response curves of acetylcholine (ACH, 0.01–100 μM) were constructed with a PHE pre-contraction (3 μM). For testing vascular reactivity, we isolated the tPVAT from 8-week-old WKY, 16-week-old WKY, 8-week-old SHR, and 16-weeke-old SHRs, respectively.

### Adenosine Measurement

Adenosine level in the conditioned medium was determined using mouse/rat adenosine ELISA kit (zc-54044, ZCI BIO). Briefly, the tPVAT was incubated in DMEM for 12 h and the medium was centrifuged at 3000 rpm for 10 min. Then the medium was immediately stored at -80°C until assaying. ELISA was performed according to the manufacturer’s instructions. A monoclonal antibody specific for mouse/rat adenosine was pre-coated onto a microplate. Standards and samples were pipetted into the wells and any adenosine present was bound by the immobilized antibody. After washing away any unbound substances, an enzyme-linked polyclonal antibody specific for mouse/rat adenosine was added to the wells. Following a wash to remove any unbound antibody–enzyme reagents, a substrate solution was added to the wells. The enzyme reaction yielded a blue product that turned yellow when the stop solution was added. The intensity of the color measured is in proportion to the amount of adenosine bound in the initial step. The sample values were then read off the standard curve. The reactions were read using an ELISA reader RT-6000 (Rayto, United States) at 450 nm. Data are expressed in pictogram per milliliter.

### Statistical Analysis

All values are given as means ± SEM. Statistical analysis was performed using one-tailed or two-tailed Student’s *t*-test. For experiments in which more than two groups were compared, two-way analysis of variant (ANOVA) was used and followed by the *post hoc* Dunnett’s test for data with more than two groups (Levene’s tests for equal variance). Dunnett’s T3 test was used for *post hoc* test comparison for the analysis of unequal variances (Welch’s and Brown–Forsythe’s test). *P*-value of 0.05 or less was deemed statistically significant in all of these statistical tests.

## Results

### Characteristics of tPVAT

**FIGURE 1:**
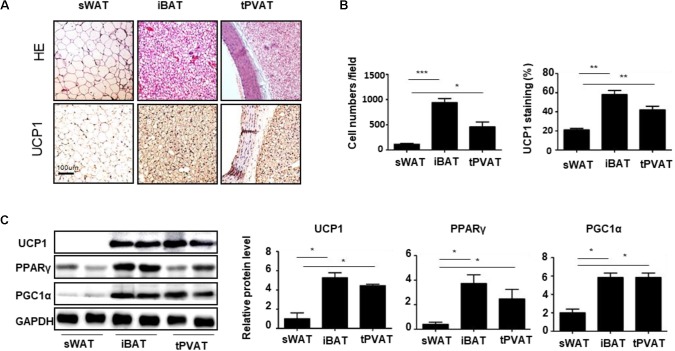
Characteristic differences among sWAT, iBAT, and tPVAT. **(A)** Representative HE and UCP1 staining images of sWAT, iBAT, and tPVAT from 8-week-old WKY rats. Magnification bar = 100 μm. **(B)** Quantitative analysis of cell numbers and UCP1 signals in sWAT, iBAT, and tPVAT from WKY rats (*n* = 4). **(C)** Western blot analysis of UCP1, PGC1α, and PPARγ protein levels in sWAT, iBAT, and tPVAT. GAPDH serves as internal control. Quantitative results are shown on the right (*n* = 4). ^∗^*p* < 0.05; ^∗∗^*p* < 0.01; ^∗∗∗^*p* < 0.001.

To explore the characteristics of tPVAT, we detected the sWAT, iBAT, and tPVAT from WKY rats. HE staining revealed that tPVAT showed a similar phenotype to iBAT, including smaller adipocyte size and increased UCP1 staining compared to sWAT (**Figures 1A,B**). Moreover, tPVAT and iBAT shared a similar thermogenic marker protein expression pattern including UCP1, PPARγ, and PGC1α (**Figure 1C**). These demonstrate that tPVAT has similar features to iBAT, whereas it is clearly different from sWAT.

### Characteristics of tPVAT Browning in SHR During Aging

**FIGURE 2:**
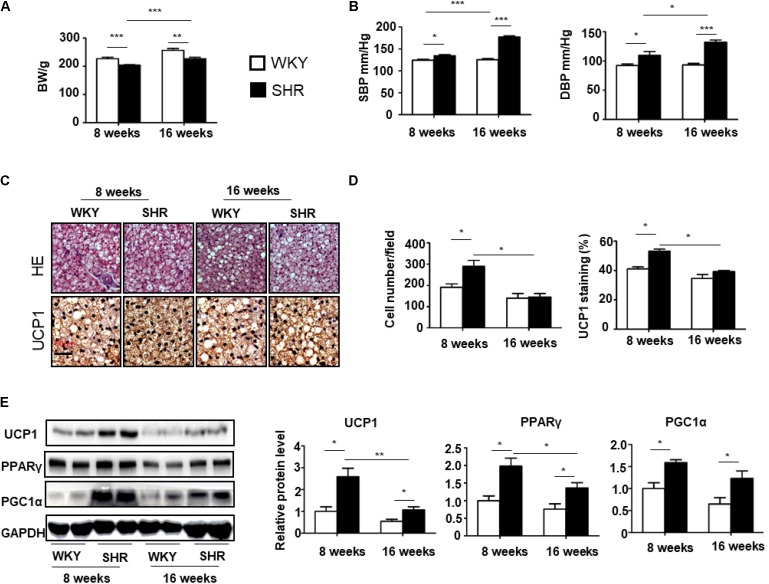
Browning level of tPVAT increases in SHR during aging. **(A)** The body weight of 8-week-old and 16-week-old WKY and SHR (*n* = 10). **(B)** The SBP and DBP of 8-week-old and 16-week-old WKY and SHR (*n* = 10). **(C)** Representative HE and UCP1 staining images of the tPVAT of 8-week-old and 16-week-old WKY and SHR. Magnification bar = 50 μm. **(D)** Quantitative analysis of cell numbers and UCP1 signals in tPVAT from 8-week-old and 16-week-old WKY and SHR rats (*n* = 4). **(E)** Western blot analysis of UCP1, PGC1a, and PPARγ expression levels in the tPVAT of 8-week-old and 16-week-old WKY and SHR. Quantitative results are shown on the right (*n* = 4). ^∗^*p* < 0.05; ^∗∗^*p* < 0.01; ^∗∗∗^*p* < 0.001.

Next, we determined the changes in the tPVAT in WKY and SHRs during aging. The body weight of WKY rats at 8 and 16 weeks of age was higher than those of SHR (227.2 ± 4.6 g in 8-week-old WKY, 203.6 ± 2.1 g in 8-week-old SHR, 256.3 ± 6.6 g in 16-week-old WKY, and 227.2 ± 4.6 g in 16-week-old SHR) (**Figure 2A**). The SBP and DBP showed a much greater increase in SHR compared with WKY at 16 weeks of age (SBP 125.3 ± 2.6 mmHg and DBP 93.1 ± 2.7 mmHg in 16-week-old WKY, SBP 177.3 ± 2.8 mmHg and 132.1 ± 3.5 mmHg in 16-week-old SHR), whereas the SBP and DBP had a slight increase in 8-week-old SHR (SBP 114.2 ± 2.4 mmHg and DBP 92.3 ± 2.6 mmHg in 8-week-old WKY, SBP 134.5 ± 2.5 mmHg and 109.4 ± 6.4 mmHg in 8-week-old SHR) (**Figure 2B**). To delineate the changes of tPVAT browning during aging in SHR, we detected expression of brown adipocyte markers in the tPVAT of 8-week-old and 16-week-old SHR and WKY rats. Compared with WKY rats, tPVAT browning activity including smaller-size adipocytes and UCP1 staining were elevated in 8-week-old SHR, but these phenotypic changes were blunted in 16-week-old SHR (**Figures 2C,D**). In accordance with these histological changes, the UCP1 and PPARγ expression showed a dramatic decrease in 16-week-old SHR compared to 8-week-old SHR, whereas UCP1, PPARγ, and PGC1α levels had no statistical difference between 8-week-old and 16-week-old WKY rats (**Figure 2E**).

### The Effects of tPVAT on Vasomotor Reaction

**FIGURE 3:**
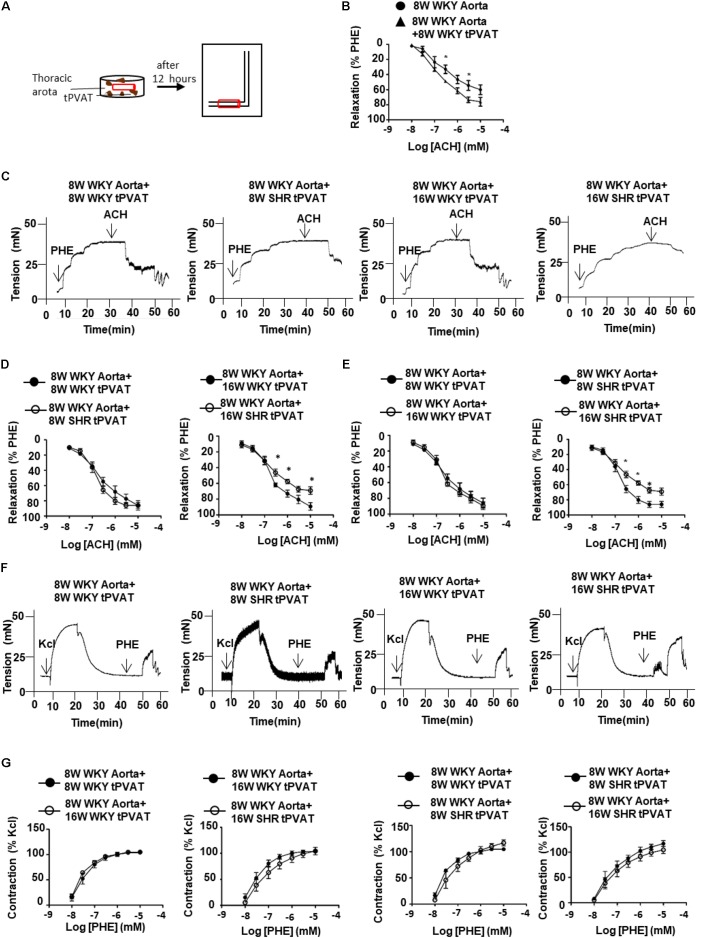
Thoracic aorta reactivity incubated with tPVAT from WKY or SHR. **(A)** Illustration of bioassay protocol. **(B)** Average percent relaxation of thoracic aorta to ACH with or without 12 h tPVAT treated (*n* = 6). **(C)** Representative raw tracing of diastolic response to ACH after PHE (3 μM) pre-contraction. The thoracic aorta rings were collected from 8-week-old WKY rats and the PVAT adjacent to the rings was cleaned. The donor tPVAT were collected from 8-week-old and 16-week-old WKY or SHR. The rings were incubated with tPVAT in DMEM for 12 h at 37°C. **(D)** Average percent relaxation to ACH of thoracic aorta treated with 8-week-old or 16-week-old WKY or SHR tPVAT (*n* = 6). **(E)** Average percent relaxation to ACH of thoracic aorta treated with 8-week-old or 16-week-old WKY or SHR tPVAT (*n* = 6). **(F)** Representative raw traces showing dose-dependent aortic ring constriction in response to PHE after KCl (60 mM) pre-contraction. **(G)** Average percent contraction to PHE of thoracic aorta treated with 8-week-old or 16-week-old WKY or SHR tPVAT (*n* = 6). ^∗^*p* < 0.05; ^∗∗^*p* < 0.01; ^∗∗∗^*p* < 0.001.

Next, to determine whether tPVAT is involved in the regulation of vascular contraction and relaxation in WKY and SHR, we utilized tPVAT to incubate thoracic aorta and analyzed the aortic vasomotor reactions (**Figure 3A**). Thoracic aortas without tPVAT from 8-week-old WKY were incubated with or without 0.3 g tPVAT from WKY or SHRs for 12 h. In aortas incubated with tPVAT, vasodilation to ACH was increased compared to those without tPVAT incubation (**Figure 3B**). Then we collected tPVAT from 8- or 16-week-old WKY and SHR to incubate thoracic aortas from 8-week-old WKY. Interestingly, the vasodilative effect had no significant difference between groups incubated with tPVAT from 8-week-old WKY and SHR. In contrast, 16-week-old WKY showed an enhanced vascular relaxation effect compared to age-matched SHR (**Figures 3C,D**). More importantly, the vasodilative effect of the tPVAT from 16-week-old SHR was significantly attenuated compared to 8-week-old SHR, whereas this effect of tPVAT had no significant difference between 16-week-old and 8-week-old WKY (**Figures 3C,E**). In addition, vasocontraction of thoracic aortas incubated with WKY and SHR tPVAT showed no significant differences at 8 or 16 weeks of age (**Figures 3F,G**). These suggest a decreased PVRF production in the tPVAT of SHRs during aging.

### The tPVAT-Derived Adenosine in SHRs

**FIGURE 4:**
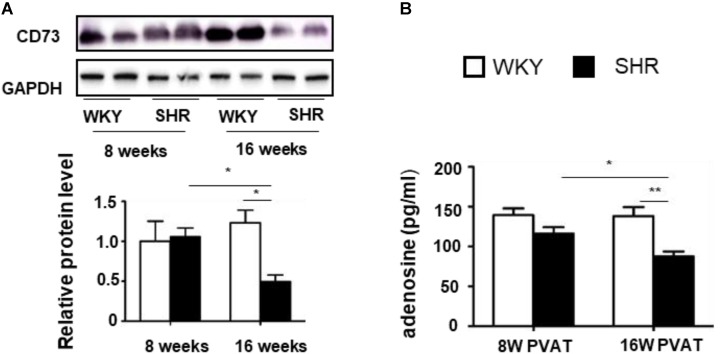
CD73 expression and adenosine production in the tPVAT of SHR decrease during aging. **(A)** Western blot analysis of CD73 protein level in the tPVAT of 8-week-old and 16-week-old WKY and SHR. Quantitative results are shown on the lower panel (*n* = 4). **(B)** Adenosine concentration (ELISA) in the tPVAT-conditional media from 8-week-old and 16-week-old WKY and SHR (*n* = 4). ^∗^*p* < 0.05; ^∗∗^*p* < 0.01; ^∗∗∗^*p* < 0.001.

A previous study showed a significant increase of adenosine in the process of BAT activation (Biswas, 2017). To determine whether tPVAT produces and releases adenosine, we firstly detected CD73 expression, which is necessary for adenosine production (Quast et al., 2017). CD73 level in the tPVAT had no significant difference between 8-week-old WKY and SHR. Interestingly, CD73 level dramatically decreased in 16-week-old SHR compared with 8-week-old SHR, whereas CD73 level had no significant changes in WKY during aging (**Figure 4A**). Consistently, adenosine concentration showed no significant difference in the tPVAT-conditioned medium of 8-week-old SHR (116.2 ± 8.0 pg/ml) and WKY (139.4 ± 8.4 pg/ml). In contrast, adenosine significantly decreased in the PVAT-conditioned medium of 16-week-old SHR (87.6 ± 6.1 pg/ml) compared with 16-week-old WKY (138.3 ± 11.3 pg/ml) or 8-week-old SHR (**Figure 4B**). These data suggest that the changes of PVAT-derived adenosine in SHR might contribute to vascular tone dysfunction.

## Discussion

This is the first study providing evidence that tPVAT browning level and related relaxing factor adenosine are decreased in SHR rats during aging. A comparison of the expression of BAT phenotypic makers in the tPVAT of 8- or 16-week-old WKY and SHRs allowed us to conclude that the browning level in SHR tPVAT is much higher than WKY, but declines quickly in SHR during aging. Accordingly, tPVAT-derived adenosine decreased from 8 to 16 weeks of age in SHR. Thoracic aorta incubated with 16-week-old SHR tPVAT showed decreased diastolic activity compared to 8-week-old SHR or 16-week-old WKY rats.

PVAT can resemble either WAT or BAT depending on the anatomical localization. Abdominal and mesenteric PVAT appears to be similar to WAT with large lipid droplets and low expression of UCP1. In contrast, tPVAT is morphologically and functionally similar to BAT (Fitzgibbons et al., 2011). These contribute to maintaining intravascular temperature during cold exposure via initiating thermogenesis. Activation of PVAT by β3-adrenergic receptor increases lipid uptake and protects against atherosclerosis (Berbee et al., 2015). Compared to WKY, the PVAT of SHR shows a smaller adipocyte size, a lower total lipid, and a decline of leptin. These may together contribute to vascular resistance and dysfunction in SHR (Li et al., 2013). Herein we provided direct evidence that the tPVAT of SHR has a higher level of BAT markers than WKY, including UCP1, PPARγ and PGC1α. Aging is associated with a ubiquitous decline of BAT activity throughout the life (Lecoultre and Ravussin, 2011). We herein first provide evidence that the browning level of SHR tPVAT shows a greater decline than WKY rats during aging from 8 to 16 weeks of age. It is well known that the blood pressure elevates quickly from 8 to 16 weeks of age in SHR rats. Our results provide a potential possibility that the earlier loss of browning in the PVAT might contribute to the blood pressure elevation in SHR rats.

Perivascular adipose tissue significantly modulates vascular contraction and relaxation via releasing PVRF and PVAT-derived contracting factor (PVCF) (Gao et al., 2006). The exact constituents of PVRF and PVCF are not yet fully understood. However, several bioactive substances have been suggested to regulate vasomotor reactions. Among these, adiponectin is the most studied PVRF. Adiponectin knockout mice or neutralizing antibody against adiponectin blocks the vasodilator response of PVAT. Obesity-induced decline of adiponectin attenuates the anti-contractile effect of PVAT (Almabrouk et al., 2018). Besides adipokines, eNOS has also been demonstrated to be expressed in PVAT. NO synthesized from eNOS is responsible for the vasodilating properties of PVAT. It is worth noting that tPVAT exhibits an increase in eNOS expression and NO production compared to abdominal PVAT (Victorio et al., 2016). On the other hand, few PVCF has been documented to regulate vascular contraction. A recent report showed that brown adipocytes in the tPVAT produce and release angiotensinogen, which contributes to the regulation of PVAT contractile property and blood pressure elevation (Chang et al., 2018). Herein, we provide the first evidence that adenosine may be a potential PVRF, which is decreased in the tPVAT of SHRs during aging. Adenosine, as a small molecular metabolite, has been demonstrated to be produced by browning adipocytes (Ammar et al., 2004). We further provide evidence of adenosine production in the tPVAT in the present study. The tPVAT has a similar vasomotor reaction and adenosine release in 8-week-old WKY and SHR rats. In accordance with loss of brown adipocytes, the tPVAT of 16-week-old SHR produces less adenosine compared with 8-week-old SHR. Consistently, the tPVAT shows attenuated diastolic effect in SHR during aging, whereas the contractive effect has no significant change. Taken together with a previous study, which showed a decreased relaxing factor methyl palmitate in the PVAT of SHR (Lee et al., 2011), these provide evidence that PVAT of SHR dysfunction might be due to the lower PVRF production. The present study provides novel evidence that the browning level of tPVAT is closely associated with vasorelaxation in hypertension.

Since rodent PVAT has been demonstrated to be histologically and functionally different compared to most types of human PVAT (Chatterjee et al., 2009), more human studies are needed to determine the accurate mechanisms by which PVAT-derived substances modulate vascular homeostasis in hypertension during aging. Although CD73-regulated adenosine release from the tPVAT affects vascular function in hypertension, the detailed mechanism and signaling pathways of this adenosine-dependent response remain to be further investigated in the future. The influence of aging on PVAT browning progress and tPVAT-derived adenosine production also needs to be documented in the future preclinical studies.

In summary, we demonstrate that aging induces a dramatic decrease of tPVAT browning in SHRs, which is associated with reduced adenosine production in tPVAT and attenuated vasorelaxing effect on the aorta. Notwithstanding the complexity of human hypertension compared to rodent models, our unique observation indicates an attractive possibility that the PVAT could serve as a therapeutic tool for preventing hypertensive vascular dysfunction during aging.

## Author Contributions

P-JG, C-CR, and L-RK designed the experiments and wrote the paper. L-RK and D-RC performed the animal experiments and analytical methods. L-RK and Y-PZ performed *in vivo* imaging. L-RK, C-CR, and P-JG analyzed the data.

## Conflict of Interest Statement

The authors declare that the research was conducted in the absence of any commercial or financial relationships that could be construed as a potential conflict of interest.
